# Takotsubo Cardiomyopathy in the Setting of Hypertrophic Cardiomyopathy Following Pacemaker Implantation

**DOI:** 10.7759/cureus.36812

**Published:** 2023-03-28

**Authors:** George Boghdadi, Emmanuel Bassily

**Affiliations:** 1 Internal Medicine, George Washington School of Medicine & Health Sciences, Washington DC, USA; 2 Cardiology, University of South Florida Morsani College of Medicine, Tampa, USA

**Keywords:** sympathetic overactivity, pacemaker complications, 3rd degree heart block, takostsubo cariomyopathy, hypertrophic obstructive cardiomyopathy (hocm)

## Abstract

The presentation of Takotsubo cardiomyopathy (TC) has overlapping features with acute coronary syndrome (ACS), though traditionally developing from different triggers, including both physical and emotional. Additionally, TC is associated with multiple comorbidities and sequelae. We present a multifactorial case of a 73-year-old female with underlying hypertrophic cardiomyopathy who presented with a high-degree atrioventricular (AV) block requiring permanent pacemaker (PPM) placement and subsequently developed TC. Patients with hypertrophic obstructive cardiomyopathy (HCM) have been theorized to have increased cardiac sympathetic activity and sensitivity. Thus, this case report demonstrates the increased relative risk of patients with underlying HCM in the development of TC during PPM placement.

## Introduction

Though Takotsubo cardiomyopathy (TC), or stress cardiomyopathy, was first described in 1991 [1], there is still a limited understanding of its etiologies, pathogenesis, associations, and effective treatments due to the paucity of cases. TC has been considered to be an acute heart failure syndrome that can acutely mimic the clinical presentation of acute coronary syndrome and is generally preceded by emotional or physical stressors [2]. Although usually reversible, TC has been associated with arrhythmias, left ventricular outflow tract (LVOT) obstruction, cardiogenic shock, mitral regurgitation, and even death [3].

## Case presentation

**Figure 1:**
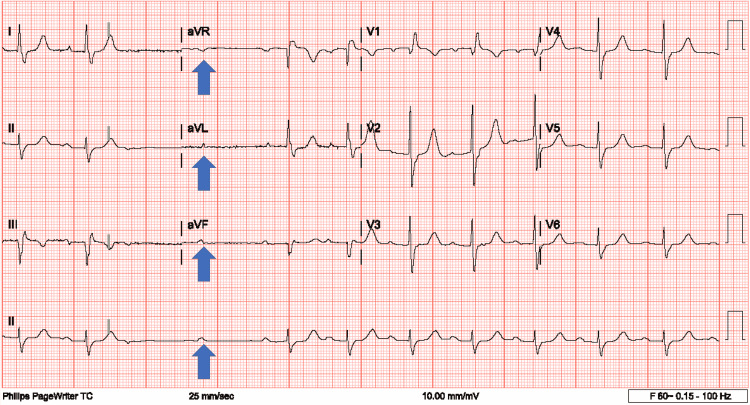
Electrocardiogram on arrival at the ED Twelve-lead electrocardiogram showing Second-degree Mobitz Type II heart block on arrival at the Emergency Department (ED). Arrows point to non-conducted p wave. The PR intervals remained stable, ruling out Wenckebach physiology

A 73-year-old female patient presented with multiple pre-syncopal episodes over the past month. She was found to be bradycardic, with high-degree atrioventricular (AV) blocks (Figure 1), and documented pauses requiring intravenous atropine.

**Figure 2:**
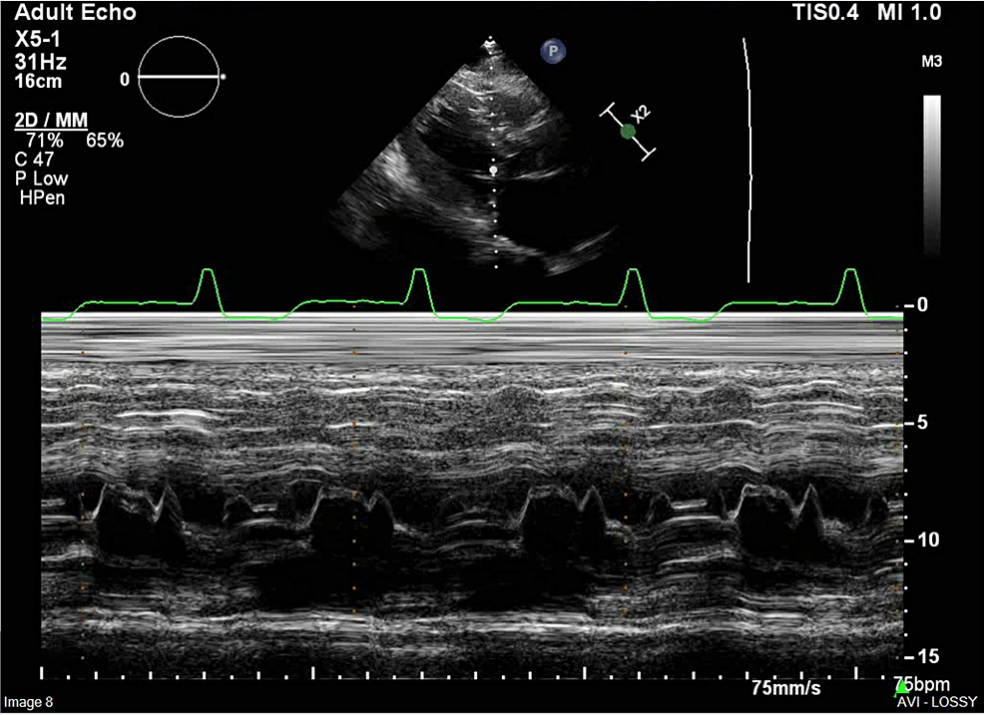
M-mode showing systolic anterior motion of mitral leaflet

Her transthoracic echocardiogram (TTE) revealed a new diagnosis of hypertrophic obstructive cardiomyopathy (HCM) and an ejection fraction (EF) of 60-65% (Video 1, Video 2, Figure 2). She underwent urgent dual chamber permanent pacemaker (PPM) implantation, and within hours, she complained of bilateral upper extremity, scapular, and cervical neck pain with associated substernal chest pressure. Subsequently, she was found to have elevated cardiac and inflammatory biomarkers: Troponin 32.6 (reference range: 0.000 - 0.028 ng/ml), B-Natriuretic peptide 7303 (reference range: <100 pg/ml), and high sensitivity CRP 17.12 (reference range: 0.01 - 0.5 mg/dl), repeat TTE showed a newly reduced EF of 25- 30% with dyskinesis of the apical myocardium, and EKG with paced rhythm and nonspecific ST segment changes. Left heart catheterization showed non-obstructive coronary artery disease confirming the diagnosis of TC.

**Video 1:** Echocardiogram with systolic anterior motion of mitral leaflet with resulting left ventricular outflow tract obstruction.

**Video 2:** Color doppler with systolic anterior motion of mitral leaflet with resulting left ventricular outflow tract obstruction and mild eccentric MR MR: Mitral regurgitation

The patient developed flash pulmonary edema and was started on gentle intravenous (IV) diuretics and eventual beta-blocker therapy. Prior to discharge, the patient had significant improvement in respiratory status and repeat TTE showed an EF of 45-50%.

## Discussion

TC is commonly seen in elderly women and classically presents with chest pain, dyspnea, or syncope [2,4]. Clinical course is usually marked by elevated troponin, elevated BNP, reduced ejection fraction, and EKG changes including ST-segment elevation, Q- or T-wave changes [2]. Although apical ballooning of the left ventricle characterizes the most common variant, other variants include midventricular, basal, and focal forms [2]. Echocardiogram of those with TC does not usually demonstrate systolic anterior motion of the mitral leaflet and subsequent left ventricular outflow tract obstruction such as those with HCM [3]. As such, TC represents about 1-2% of those presenting with acute coronary syndrome (ACS) [5]. However, coronary angiography usually reveals non-obstructive coronary artery disease and remarkable recovery over time in TC [1]. Thus, TC is a diagnosis of exclusion. 

Recent literature has revealed that emotional triggers are not as common as physical triggers as inciting events in the development of TC [2,4]. Emotional triggers can include both positive and negative events (e.g. birth of a family member or death of a family member) [4]. These physical triggers include illnesses and medical procedures [4]. Our patient required PPM placement given her high degree AV heart block and subsequently developed TC. Similarly, there have been a few case reports demonstrating this phenomenon [6]. Although the etiology of TC is still unclear, it is recognized that increased sympathetic nervous system (SNS) activity and the release of catecholamines play a central part in disease pathogenesis [7]. Working within this schema, we propose that PPM implantation acts as a physical trigger for the development of TC secondary to increasing SNS activity. Patients with HCM have been theorized to have increased cardiac sympathetic activity and sensitivity [8]. Given this patient's HCM, she may theoretically be at higher risk for developing TC. HCM may be a risk factor for the development of TC in the setting of medical procedures in this specific patient population.

## Conclusions

Although many components of the above case have been recorded in the literature individually, the multifactorial nature of the above patient’s presentation remains relatively unique in regard to diagnosis and management. This case illustrates the overlapping features of presentation between ACS and TC. Our literature review also reveals that TC as a complication of PPM, though rare, should be taken into consideration among those at risk. This case report reveals that HCM may also be a predisposing factor in the development of TC, especially in the setting of physical triggers.
